# Validation of continuous intragastric pressure measurement and correlation with intramucosal pH in a pig model

**DOI:** 10.1186/cc9499

**Published:** 2011-03-11

**Authors:** M Malbrain, I De laet, L Luis, L Correa, M Garcia, G Castellanos

**Affiliations:** 1 ZNA Stuivenberg, Antwerp, Belgium; 2 Neuron NPh, SA, Granada, Spain; 3 Jesús Usón Minimally Invasive Surgery Center, Cacères, Spain; 4 'Virgen de la Arrixaca' University Hospital, Murcia, Spain

## Introduction

The aim of this study was the validation of continuous intragastric pressure (IGP) measurement and correlation with intramucosal pH (pHi) in a pig model of intra-abdominal hypertension (IAH).

## Methods

In 51 pigs, 611 paired IAP measurements were performed. IAP was measured at end-expiration using two different methods: the gold standard via an indwelling bladder catheter (IVP), and via a balloon-tipped nasogastric tube (IGP). During the same period 86 simultaneous pHi and IGP measurements were performed in 40 pigs. The abdominal perfusion pressure (APP) was defined as mean arterial pressure (MAP) minus IAP. Statistical analysis was done via Pearson correlation and Bland-Altman analysis; values are mean ± SD unless stated otherwise.

## Results

Mean IGP was 22.3 ± 12.7 mmHg (range 0 to 43.1), and IVP was 22.9 ± 12.6 (0 to 48). There was a very good correlation between IGP and IVP. For the whole set of paired measurements (*n *= 611), IVP = 1.02 × IGP (*R*^2 ^= 0.96, *P *< 0.0001); and for the means per individual pig (*n *= 51), IVP = 1.03 × IGP (*R*^2 ^= 0.96, *P *< 0.0001). The analysis according to Bland-Altman for the whole set (*n *= 611) showed a mean IAP of 22.6 ± 12.6 (0.1 to 44) with a bias (±1.96 × SD) of 0.6 ± 2.4 mmHg; the limits of agreement (LA) were -4.2 to 5.5 mmHg (% error of 21.5). Looking at the mean values in each individual animal mean IAP was 22 ± 9.4 (2.5 to 37.9), with a bias of 0.8 ± 1.9 (LA -3 to 4.6) and a % error of 17.2. These intervals are small and reflect a good agreement between the two IAP methods. The mean pHi was 7.02 ± 0.28 (6.34 to 7.37) and correlated well with IGP (*R*^2 ^= 0.7, *P *< 0.001). Analysis further showed that changes in IGP correlated well with changes in pHi (*R*^2 ^= 0.66, *P *< 0.001). The MAP was 48.3 ± 14 (3 to 138) and APP was 24.9 ± 17.4 (0.2 to 92). During 388 paired measurements APP correlated significantly with pHi (in a logarithmic fashion, *R*^2 ^= 0.18), the correlation was linear and even better in conditions when APP <45 mmHg (*n *= 334): pHi = 0.016 × APP + 6.63 (*R*^2 ^= 0.55, *P *< 0.0001). Thus increased APP above 45mmHg did not result in a further increase of pHi.

## Conclusions

We found a very good correlation between IGP and IVP. Measurement via the stomach has major advantages over the standard intravesical method: continuous measurement of IAP as a trend over time is possible and there is no interference with estimation of urine output. Moreover, APP is correlated with pHi while IAP and pHi are inversely correlated.

